# Is Mechanical Power the Trojan Horse? Emphasizing the Role of Driving Pressure

**DOI:** 10.3390/medicina61122086

**Published:** 2025-11-22

**Authors:** Dicle Birtane, Furkan Tontu, Zafer Çukurova, Sinan Aşar

**Affiliations:** 1 Department of Anesthesiology and Reanimation, Bakirkoy Dr. Sadi Konuk Training and Research Hospital, Istanbul 34147, Turkey; dicle1tane@gmail.com (D.B.); zcukurova@gmail.com (Z.Ç.); 2 Department of Anesthesiology and Reanimation, Basaksehir Cam and Sakura City Hospital, Istanbul 34480, Turkey; 3 Department of Reanimation, Mardin Research and Training Hospital, Mardin 47100, Turkey; sinan.asaras@gmail.com

**Keywords:** driving pressure, mechanical power, acute respiratory distress syndrome, prognosis, mortality, COVID-19

## Abstract

*Background and Objectives*: Driving pressure (DP) and components of mechanical power (MP) have been proposed as prognostic markers in ARDS. The prognostic significance of these parameters in COVID-19–associated ARDS (C-ARDS), particularly during the early phase of intensive care unit (ICU) stay, remains uncertain. *Materials and Methods*: A retrospective single-center cohort of 310 C-ARDS patients receiving invasive mechanical ventilation was analyzed. Ventilator data from the first 72 h after ICU admission were retrieved. DP, total mechanical power (MPtot), and dynamic mechanical power (MPdyn) were calculated. The primary endpoint was defined as ICU mortality; secondary endpoints were ventilator-free days (VFDs) and length of stay (LOS) in ICU. ROC analyses, Cox proportional hazards regression, and Kaplan–Meier survival estimates were applied. *Results:* DP ≥ 15.72 cm H_2_O and MPdyn ≥ 10.08 J/min were found to be significantly associated with increased ICU mortality (HR 1.9 [1.5–2.5], *p* < 0.0001; HR 1.5 [1.2–1.9], *p* = 0.0036, respectively), whereas MPtot ≥ 18.6 J/min was not (*p* = 0.1). Patients with DP below the threshold demonstrated longer VFDs, and higher survival probabilities. No significant differences in VFDs were identified for MPdyn or MPtot. *Conclusions*: In C-ARDS patients, early measurements of DP and MPdyn were independently associated with ICU mortality, while MPtot was not. Among these parameters, DP may be regarded as the most practical marker due to its ease of calculation and potential utility in guiding lung-protective ventilation strategies.

## 1. Introduction

Lung-protective mechanical ventilation remains the cornerstone of acute respiratory distress syndrome (ARDS) management, with the primary aim of minimizing ventilator-induced lung injury (VILI) and improving clinical outcomes [1,2,3]. Among ventilatory parameters, driving pressure (DP)—defined as the difference between plateau pressure and PEEP—has emerged as an important predictor of mortality in ARDS [4]. DP has been defined as a critical indicator, reflecting the stress applied to the functional portion of the lung, and has since gained broad clinical significance [5].

In ARDS patients, the prognostic value of DP has been demonstrated, with values exceeding 15 cm H_2_O being associated with increased mortality [6]. However, its clinical significance in the C-ARDS population remains under investigation [7,8]. In addition, whether mechanical power (MP)—a comprehensive parameter integrating tidal volume, respiratory rate, and airway resistance—provides incremental prognostic value beyond DP is still being explored [9,10]. While total mechanical power (MP_tot_) has been linked to VILI in preclinical studies, recent investigations suggest that isolating dynamic mechanical power (MP_dyn_), which excludes the contribution of baseline PEEP, may reveal physiologically more meaningful associations [11,12].

In this study, the relationship between DP, MP_dyn_, and MP_tot_ and clinical outcomes was investigated in a well-defined cohort of patients undergoing mechanical ventilation due to C-ARDS. Continuous ventilator data from the first 72 h of intensive care unit (ICU) stay were used to assess the prognostic value of these parameters. The primary outcome was ICU mortality, while secondary outcomes included ventilator-free days (VFDs) and length of stay (LOS) in ICU.

## 2. Materials and Methods

### 2.1. Ethical Approval

This study was approved by the Ethics Committee of Bakırköy Dr. Sadi Konuk Training and Research Hospital (Protocol No: 2021/471, Decision No: 2021-20-06). At the time of admission to the intensive care unit (ICU), the relatives of the patients were informed in detail that patient data could be used retrospectively for scientific research, and written informed consent was obtained from them.

### 2.2. Study Design and Data Collection

**Figure 1:**
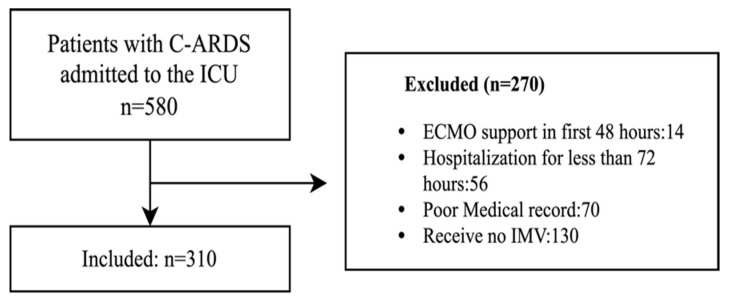
Flow chart of the study. ARDS: Acute Respiratory Distress Syndrome, C-ARDS: COVID-19–Associated ARDS, ECMO: Extracorporeal Membrane Oxygenation, ICU: Intensive Care Unit, IMV: Invasive Mechanical Ventilation.

Data from 580 COVID-19 patients admitted to the ICU of Bakırköy Dr. Sadi Konuk Training and Research Hospital between 18 March 2020, and 1 October 2021, were retrieved using SQL queries from the ImdSoft-Metavision/Q_lin_ICU Clinical Decision Support System (Canada). Of these, 310 patients diagnosed with ARDS according to the Berlin criteria—168 of whom were PCR-positive, with all demonstrating CT findings consistent with ARDS—were included in the study (Figure 1).

### 2.3. Parameters

#### 2.3.1. Demographic Parameters

Age, sex, height, predicted body weight (PBW), body mass index (BMI), SOFA and APACHE II scores at ICU admission were extracted via SQL queries and exported to Excel.

#### 2.3.2. Hemodynamic Parameters

Minute-by-minute values for heart rate (HR), systolic arterial blood pressure (ABP Sys), diastolic arterial blood pressure (ABP Dias), and mean arterial pressure (ABP Mean) were obtained from the database using SQL queries and exported to Excel.

#### 2.3.3. Respiratory Parameters

All orotracheally intubated patients were ventilated using Maquet Servo-i (Sweden) ventilators in either pressure control (PCV) or volume control (VCV) modes. Respiratory parameters—including peak pressure (P_peak_), airway inspiratory pressure, DP, PEEP, mean airway pressure [Pmean; for PCV: ((P_peak_ − PEEP) × (T_insp_/T_total_)) + PEEP; for VCV: ((P_peak_ − PEEP) × ½ × (T_insp_/T_total_)) + PEEP], respiratory rate (RR), tidal volume (TV), compliance (C), work of breathing (WOBv), and inspiratory-to-expiratory ratio (I:E ratio)—were collected under deep sedation during controlled PCV or VCV ventilation.

In patients receiving fully controlled mechanical ventilation under deep sedation, ventilatory and hemodynamic parameters were automatically recorded on a minute-by-minute basis by the clinical information system (Metavision back server). Raw data were extracted via SQL queries, and periods involving aspiration, circuit disconnection, alarm activation, or ventilator maneuvers were excluded based on system flags to eliminate artifact-containing measurements from the analysis. Due to the high frequency of data points (4320 min per patient × 310 patients = 1,339,200 rows) and to reduce the effect of short-term physiological fluctuations, minute-based values were first converted into hourly mean values. Subsequently, mean values for the first 72 h were calculated for each patient to obtain representative ventilatory and hemodynamic parameters.

Among the patients included in the study, 98% were ventilated in PCV mode and 2% (6 patients) in VCV mode.

In VCV, the ventilator default setting includes a Tpause of 10%. At this setting, the Servo-i ventilator automatically measures the plateau pressure (P_plateau_) without requiring an inspiratory hold maneuver. Subsequently, when the expiratory flow (V˙_ee_) reaches zero, the ventilator automatically measures total PEEP. These data are recorded minute-by-minute on the Metavision server. Driving pressure (DP) is automatically calculated on the server as P_plateau_ − total PEEP.

In PCV mode, airway pressure was considered constant at the end of inspiration, and peak inspiratory pressure (P_peak_) and alveolar pressure (P_plateau_) were assumed to be equal (P_plateau_ = P_peak_) [13,14,15,16].

ΔP_insp_ = P_plateau_ − PEEP = P_peak_ − PEEP

If intrinsic PEEP is absent, compliance (C) can be calculated as:

C = ΔV/ΔP_insp_

It is known that, in PCV, four times the expiratory time constant (4τ) is required for exhalation of 98% of the inspiratory volume to eliminate intrinsic PEEP [16]. In addition, end-expiratory flow (V˙_ee_) is expected to approach zero [17]. In ARDS patients ventilated in PCV mode, inspiratory resistance (Ri) and expiratory resistance (Re) are similar according to the single-compartment model, and resistance is considered equal during inspiration, expiration, and the entire respiratory cycle [14,18]. Arnal et al. reported R = 15 cm H_2_O·s/L in ARDS patients using passive humidification in PCV mode [17].

τ = R × C

Thus, the four time constant can be expressed as 4τ = 60 × C for an average resistance of 15 cm H_2_O·s/L under PCV.

The total respiratory cycle duration was calculated as T_total_ = 60/RR = T_insp_ + T_exp_ and expiratory time as T_exp_ = T_total_ − T_insp_ [17].

The 4τ values and expiratory times (T_exp_) were compared using a paired *t*-test. The mean and SD of the differences were 0.65 ± 0.32 s (*p* < 0.0001), indicating that the expiratory time was significantly longer than 4τ. In addition, the mean ± SD of end-expiratory flow (V˙_ee_) across 4320 min of data from 310 patients was 0.01 ± 0.006 L/min. Since expiratory time was sufficiently long and V˙_ee_ approached zero, the presence of intrinsic PEEP was considered unlikely [17].

#### 2.3.4. Calculation of Length of Stay in the ICU and Ventilator-Free Days (VFDs)

The length of stay in the ICU was calculated by the software as the time between the time point when the patient was intubated in the ICU (T_admission_) and the time point of death or discharge in the ICU (T_death_ or T_discharge_).

The length of stay in ICU = T_death_ or T_discharge_ − T_admission_

At a fixed time in ICU (for 28-day mortality), the VFDs are calculated as [19,20]:

- If the patient dies while on mechanical ventilation, VFD = 28-time on mechanical ventilation (x) = 0.
- If the patient is successfully recovered from ventilation x days after being connected to the mechanical ventilator, VFD = 28 − x.
- If the patient is mechanically ventilated for >28 days, VFD = 0.

Since in this study, the duration of stay in the ICU was used instead of a fixed time (such as 28 or 30 days) for mortality estimation, the VFD’s were calculated as follows: The ventilator free minute was accepted as NULL for respiratory mechanics transferred from mechanical ventilators to the clinical decision support system between the time when the patient was intubated in the ICU (T1) and the time between death in the ICU or discharged (T2) (confirmed by nurse and doctor notes). All time intervals were recorded by the software in minutes. These minute-based time slices were transferred from the data pool to Microsoft Excel as hourly time intervals using SQL queries and subsequently converted to daily values by dividing by 24.

### 2.4. Calculation of Mechanical Power

#### 2.4.1. Calculation of Total Mechanical Power

Since orotracheally intubated patients were ventilated with VCV and PCV modes under deep sedation, the recorded values were considered representative of minute ventilation. Total mechanical power (MP_tot_) values were calculated using predefined practical equations: the volume control equation (MP_vcv-simpl_ = 0.098 × RR × TV × (P_peak_ − DP/2)) and the pressure control equation (MP_pcv-simpl_ = 0.098 × RR × TV × (PEEP + ΔP_insp_)) [9,10].

#### 2.4.2. Calculation of Dynamic Mechanical Power

Mechanical power consists of three components: elastic (DP), resistive (P_peak_ − P_plat_), and PEEP-related components [14]. The combination of the elastic and resistive components is referred to as dynamic power [15,16].

Dynamic mechanical power (MP_dyn_) is calculated as follows

For VCV: MP_dyn(vcv)_ = 0.098 × RR × TV × ((P_peak_ − DP/2) − PEEP)

For PCV: MP_dyn(pcv)_ = 0.098 × RR × TV × ΔP_insp_

### 2.5. Statistical Analysis

The normality of the variables examined in the study was assessed using the Shapiro–Wilk test. Since the patients’ data were not normally distributed, the Mann–Whitney U test was used to compare the survival and non-survival groups. Frequency distributions and percentages of categorical variables, such as gender, were compared using the Chi-square test. Median values with interquartile ranges (IQRs) were used for descriptive statistics. Statistical significance was set at *p* < 0.05. For each day and each parameter (DP, MP_dyn_, MP_tot_), Receiver Operating Characteristic (ROC) curve analyses were performed using the scikit-learn machine learning library in Python 3.10 [21]. The area under the curve (AUC) was calculated for the generated ROC curves. Logistic regression models were fitted using the statsmodels Python 3.10 module [22]. The 95% confidence intervals of the AUC values were estimated by bootstrapping. The optimal cut-off values for each parameter on the selected day were determined by identifying the threshold that maximized Youden’s Index. Patients were stratified into two groups according to the cut-off values: greater than or equal to the cut-off (GE) and less than the cut-off (LESS). Survival analysis was performed using the lifelines package in Python [23]. Kaplan–Meier survival curves were constructed for each group, with 95% confidence intervals calculated. Analyses were performed for length of ICU stay (LOS, days), duration of invasive mechanical ventilation (MV, days), and ventilator-free days (VFDs). Median survival time in the ICU (the “half-life” of the patient population, defined as the time at which 50% of patients had died) was reported for each group. To evaluate the impact of the studied parameters on survival, Cox proportional hazards regression models were applied to the grouped parameters. In addition to *p* values and 95% confidence intervals, hazard ratios (HRs) were calculated for each parameter.

## 3. Results

### 3.1. Patient Demographics and Hemodynamic Data

This study included 310 patients admitted to the ICU for C-ARDS. At ICU admission, 91 (29.4%) patients had mild C-ARDS, 163 (52.6%) had moderate C-ARDS, and 56 (18.0%) had severe C-ARDS. The median PaO_2_/FiO_2_ (P/F) values of mild, moderate, and severe C-ARDS were 243, 152, and 80, respectively. Demographic variables (gender, age, weight, height, BMI, predicted body weight, ideal weight, APACHE II, and SOFA scores) reflect measurements obtained at ICU admission, whereas the hemodynamic parameters (heart rate, ABP_sys_, ABP_dias_, ABP_mean_) represent the 72 h averaged values. The detailed patient characteristics are presented in Table 1.

### 3.2. Respiratory Parameters

Median DP values were significantly lower in survivors compared with non-survivors (15.3 [14.8–16.0] vs. 17.0 [15.6–18.8] cm H_2_O, *p* = 0.0001; Table 2). Similarly, survivors had significantly lower MP_dyn_, P_peak_, P_plat_, P_mean_, WOBv, RR, F_i_O_2_, and E_t_CO_2_, and higher TV, TV/PBW, P/F ratio, C and SpO_2_ compared with non-survivors (all *p* < 0.05, Table 2). No significant differences were observed between the groups for MP_tot_, PEEP, MVe, I/E ratio, or PaO_2_. All respiratory parameters presented in Table 2 represent 72 h averaged values.

### 3.3. Laboratory Parameters

Several laboratory parameters obtained at ICU admission significantly differed between survivors and non-survivors. Non-survivors had higher WBC (*p* = 0.0002), lower %Lym (*p* = 0.0001), higher Neu (*p* = 0.0001), higher %Neu (*p* = 0.0001), elevated procalcitonin (*p* = 0.0061), LDH (*p* = 0.0093), and creatinine levels (*p* = 0.0001). Additionally, albumin was slightly lower in non-survivors (*p* = 0.0303), and INR was significantly elevated (*p* = 0.0109). No statistically significant differences were observed in proBNP, D-dimer, ferritin, CRP, fibrinogen, electrolytes (Na, K, Cl), or several other hematological markers (*p* > 0.05) (Table 3).

Inflammatory biomarkers were compared between patients above and below the driving pressure threshold. Among these markers, WBC and Lym% differed significantly between the groups. Patients with DP ≥ 15.72 cmH_2_O had higher WBC values compared with those below the threshold (median [IQR]: 15.6 [11.8–21.3] vs. 13.0 [9.3–18.7], *p* = 0.049), along with a significantly lower Lym% (5.3% [3.6–8.4] vs. 7.4% [5.1–9.3], *p* = 0.0069). No significant differences were observed in CRP, Lym, procalcitonin, or ferritin levels between the groups (all *p* > 0.05) (Table S1).

### 3.4. Predictive Value of Ventilatory Parameters

**Figure 2:**
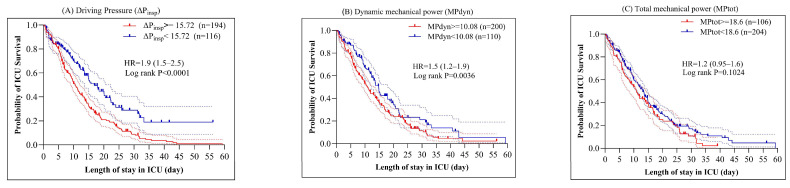
Cox regression and Kaplan–Meier survival curves showing ICU survival probability based on patient averages of driving pressure (DP = ΔP_insp_), dynamic mechanical power (MP_dyn_), and total mechanical power (MP_tot_) during the first 72 h in the ICU. Patients were grouped by whether their values were above (≥) or below (<) the determined cut-off thresholds. Dashed lines indicate the 95% confidence intervals.

ROC analysis of mean values during the first 72 h yielded an AUC (95% CI) of 0.75 (0.69–0.81) for DP, 0.62 (0.55–0.69) for MP_dyn_, and 0.56 (0.48–0.63) for MP_tot_. Optimal cut-off values determined by Youden’s Index were DP: 15.72 cmH_2_O, MP_dyn_: 10.08 J/min, MP_tot_: 18.6 J/min. Patients above the cut-off for DP and MP_dyn_ had significantly higher ICU mortality (HR 1.9 [1.5–2.5], *p* < 0.0001; HR 1.5 [1.2–1.9], *p* = 0.0036, respectively; Figure 2). By contrast, MP_tot_ ≥ 18.6 J/min was not associated with mortality (HR 1.2 [0.95–1.6], *p* = 0.1024).

Several parameters demonstrated significant associations with the length of stay in the ICU. Higher driving pressure (HR 1.85, *p* = 0.0001), elevated dynamic mechanical power (HR 1.397, *p* = 0.019), increased plateau pressure (HR 1.393, *p* = 0.013), and higher APACHE II (HR 2.111, *p* = 0.0001) and SOFA scores (HR 1.761, *p* = 0.0001) were associated with shorter LOS in ICU, reflecting earlier mortality rather than faster clinical recovery. In contrast, higher BMI (HR 0.595, *p* = 0.0001) and higher PEEP (HR 0.588, *p* = 0.0001) were associated with prolonged LOS in the ICU, reflecting slower clinical recovery (Table S2).

### 3.5. Ventilator-Free Days

**Figure 3:**
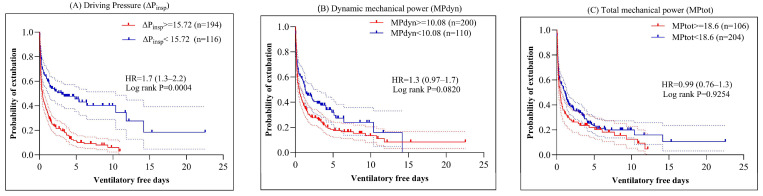
Cox regression and Kaplan–Meier survival curves showing the probability of extubation (ventilator-free days) based on patient averages of driving pressure (DP = ΔP_insp_), dynamic mechanical power (MP_dyn_), and total mechanical power (MP_tot_) during the first 72 h in the ICU. Dashed lines indicate the 95% confidence intervals.

Patients with DP <15.72 cm H_2_O had significantly longer ventilator-free days (median 2.0 vs. 0.96 days; HR 1.7 [1.3–2.2], *p* = 0.0004; Figure 3). No significant difference was observed in ventilator-free days for MP_dyn_ or MP_tot_ above and below their respective cut-offs (*p* = 0.0820 and *p* = 0.9264). In Figure 3, this outcome is displayed as the “probability of extubation,” representing the likelihood of becoming ventilator-free during the ICU stay.

## 4. Discussion

In this retrospective study of 310 patients with C-ARDS receiving invasive mechanical ventilation, elevated DP and MP_dyn_ measured within the first 72 h following ICU admission were found to be significantly associated with higher ICU mortality. In contrast, no statistically significant association was observed between MP_tot_ and mortality. Our findings indicate that both DP and MP_dyn_ are clinically important parameters for early prognostic assessment in patients with C-ARDS.

DP is increasingly recognized as one of the most reliable parameters for predicting mortality in ARDS [6,24]. It has been shown that DP is more strongly associated with patient outcomes than either TV or P_plat_ [6]. Similarly, a meta-analysis reported that DP has a decisive impact on survival [25]. In the present study, patients with DP ≥ 15.72 cm H_2_O had significantly higher ICU mortality, nearly doubling the risk. These findings are consistent with current recommendations advocating that DP be maintained below 15 cm H_2_O in lung-protective ventilation strategies [26,27]. Recent evidence indicates that even lower driving pressure values (10 cm H_2_O) are associated with reduced 30-day mortality [28].

The clinical impact of the elastic, resistive, and PEEP-dependent components of MP may differ [29]. In this study, a significant association was found between MPdyn—which excludes the effect of PEEP—and mortality, whereas no such relationship was observed for MP_tot_. However, Serpa Neto et al. demonstrated that elevated MP levels are associated with increased mortality in critically ill patients [30]. In a COVID-19–specific cohort, Schuijt et al. reported a relationship between MP and 28-day mortality [31]. Most of these studies, however, focused exclusively on MP_tot_. By isolating MP_dyn_, our study more clearly demonstrated the effect of dynamic stress on patient outcomes, suggesting that it may represent a more precise target in clinical practice. This finding supports the physiological principle that excessive cyclical mechanical stress, rather than baseline pressure, drives parenchymal injury [11].

In a recently published multicenter study, elastic power—the most important component of dynamic power (elastic + resistive)—was reported to be the parameter most strongly associated with the presence and severity of ARDS [32]. In that study, elastic power values ≥ 4.8 J/min were linked to higher ARDS severity [32]. These findings underscore that not only the magnitude but also the individual components of the energy delivered to the lungs carry clinical significance. This challenges the classical paradigm of “higher MP = greater injury” and suggests the need for a more nuanced interpretation. Our results are in line with this perspective, demonstrating that MP_dyn_, rather than MP_tot_, carried prognostic value and that the contribution of PEEP may confound the interpretation of total power. We therefore consider DP and MP_dyn_ to represent the true injurious forces, concealed within the Trojan horse of MP_tot_.

In patients with DP values below the threshold of 15.72 cm H_2_O, significantly better outcomes were observed in terms of ventilator-free days (VFDs), indicating more rapid recovery and successful liberation from mechanical ventilation. These findings highlight DP as a strong prognostic marker not only for mortality but also for functional outcomes such as ventilator dependence. In contrast, no significant differences in VFDs were observed between patients stratified by the predefined thresholds for MP_dyn_ and MP_tot_. Of note, LOS in ICU was shorter in the high-DP, high-MP_dyn_, and high-MPtot groups, which predominantly consisted of non-survivors. This shorter LOS reflects earlier mortality rather than faster clinical improvement.

Excessive mechanical strain during mechanical ventilation (e.g., high DP) has been associated with an enhanced systemic inflammatory response [33,34]. High ventilatory stress has been linked to increased leukocyte counts, ferritin concentrations, and neutrophil-to-lymphocyte ratios, indicating heightened inflammatory activation [35,36]. In our cohort, patients above the DP threshold demonstrated higher WBC levels and lower lymphocyte percentages, while CRP, absolute lymphocyte count, procalcitonin, and ferritin values did not differ significantly between groups.

In addition, older age, lower BMI, higher APACHE II and SOFA scores, and elevated P_peak_ values were found to be associated with longer ICU length of stay. These findings are consistent with the existing literature, which indicates that disease severity and impaired respiratory mechanics prolong the course of critical illness in ARDS patients [37].

This study has several limitations. Its retrospective and single-center design may limit generalizability. Given the observational design of this study and the absence of randomized controlled trials evaluating different driving pressure targets, the findings demonstrate association rather than causation. Therefore, no causal inference can be made regarding the effect of driving pressure on clinical outcomes. Transpulmonary pressure could not be measured due to the unavailability of the required equipment. The equations used for calculating MP did not incorporate direct measurements such as respiratory system compliance or esophageal pressure. Supportive interventions such as prone positioning or corticosteroid use were not separately evaluated in the regression models. In PCV mode, plateau pressure was not measured using an inspiratory hold maneuver. Since interleukin (IL) and erythrocyte sedimentation rate (ESR) values were not measured in the patients included in the study, the relationship between dynamic strain and inflammation could not be clearly demonstrated.

## 5. Conclusions

In conclusion, in patients with C-ARDS, DP and MP_dyn_ measured early after ICU admission were independently associated with mortality, whereas MP_tot_ was not. Among these parameters, DP—owing to its ease of calculation—may serve as a reliable guiding parameter in clinical decision-making.

## Figures and Tables

**Table 1:** Demographic characteristics of patients are presented as median values with interquartile ranges (IQR 25–75).

	All Patients (n: 310)	Survival (n: 76)	Non-Survival (n: 234)	*p*-Value
**Gender, Female n (%)**	124 (%40)	31 (%40.8)	93 (%39.7)	0.8
**Age (years)**	60 (47–70)	53 (43–64)	61 (50–71)	0.0008
**Weight (kg)**	80 (70–90)	83 (75–90)	80 (70–90)	0.0014 *
**Height (m)**	1.7 (1.64–1.75)	1.7 (1.65–1.8)	1.7 (1.6–1.8)	0.074
**BMI (kg/m^2^)**	27.3 (24.7–29.4)	28.2 (26–31.2)	27.2 (24.7–29.4)	0.0039 *
**PBW (kg)**	66 (56–70)	66 (57–73)	66 (56–70)	0.1030
**Ideal Weight (kg)**	75 (67–80)	76 (68–81)	75 (67–80)	0.1419
**Apache II**	23 (19–28)	20 (16–24)	25 (20–29)	0.0001 *
**SOFA**	11 (8–13)	9 (7–12)	11 (9–14)	0.0001 *
**Length of Stay in ICU (day)**	10.0 (5.7–17)	13.2 (7.7–20)	9.6 (5.1–15.5)	0.0007 *
**Invasive MV Time (day)**	8.9 (4.6–14.6)	9.7 (4.6–14.8)	8.8 (4.6–14.1)	0.2469
**Ventilator-Free Days**	0.6 (0.2–2.3)	3.2 (1.7–6.0)	0.3 (0.1–1.2)	0.0001 *
**HR (/min)**	86 (73–99)	76 (67–87)	89 (75–104)	0.0001 *
**ABP Sys (mmHg)**	120 (112–128)	122 (115–133)	120 (111–127)	0.0191 *
**ABP Dias (mmHg)**	61 (54–68)	64 (59–71)	59 (53–66)	0.0001 *
**ABP Mean (mmHg)**	80 (74–86)	84 (77–90)	79 (72–85)	0.0001 *

* *p* < 0.05. ABP Dias: Diastolic arterial pressure, ABP Mean: Mean arterial pressure, ABP Sys: Systolic arterial pressure, APACHE II: Acute Physiology and Chronic Health Evaluation II, BMI: Body mass index, HR: Heart rate, ICU: Intensive care unit, IQR: Interquartile range, MV: Mechanical ventilation, PBW: Predicted body weight, SOFA: Sequential Organ Failure Assessment.

**Table 2:** Analysis of respiratory parameters is presented as median values with interquartile ranges (IQRs).

	All Patients (n: 310)	Survival (n: 76)	Non-Survival (n: 234)	*p*-Value
**DP (cmH_2_O)**	16.4 (15.2–18.3)	15.3 (14.8–16.0)	17.0 (15.6–18.8)	0.0001 *
**MPdyn (J/min)**	11.1 (9.5–12.8)	10.2 (8.7–11.8)	11.4 (9.8–13.1)	0.0010 *
**MPtot (J/min)**	17.3 (14.8–19.5)	16.5 (14.5–18.4)	17.6 (15–19.6)	0.0623
**TV/PBW**	7.8 (7.1–8.6)	8.0 (7.2–9.0)	7.8 (7.1–8.5)	0.0194 *
**RR (/min)**	14.8 (13.8–15.9)	14 (13–15)	15 (14–16)	0.0012 *
**PEEP (cmH_2_O)**	8.7 (7.9–9.9)	9.0 (7.9–10.0)	8.7 (7.9–9.8)	0.1971
**Ppeak (cmH_2_O)**	26.3 (24.4–29.0)	25.1 (23.9–28.0)	26.8 (25–29)	0.0020 *
**Pplat (cmH_2_O)**	25.6 (23.8–27.6)	24.7 (23.4–25.5)	26.1 (24.2–28.2)	0.0001 *
**Pmean (cmH_2_O)**	15.8 (13.9–17.6)	15.3 (13.8–16.4)	16.0 (14–17.7)	0.0218 *
**TV (mL)**	488 (447–538)	509 (478–565)	483 (440–531)	0.0005 *
**MVe (mL/kg)**	7.1 (6.3–7.8)	7.2 (6.4–7.8)	7.0 (6.2–7.7)	0.1158
**C (mL/cmH_2_O)**	29.7 (24.1–34.7)	33.2 (26.8–37.8)	28.3 (22.1–31.9)	0.0005 *
**WOBv (J/L)**	1.4 (1.3–1.5)	1.3 (1.2–1.4)	1.4 (1.3–1.6)	0.0001 *
**I/E**	0.8 (0.6–1.0)	0.8 (0.5–0.95)	0.8 (0.6–1.0)	0.2145
**FiO_2_ (%)**	56 (49–66)	53 (47–61)	59 (49–68)	0.0104 *
**PaO_2_ (mmHg)**	90 (75–103)	93 (83–104)	88 (75–103)	0.0691
**P/F**	167 (120–215)	173 (139–227)	164 (118–208)	0.0221 *
**SpO_2_ (%)**	95 (92–97)	95 (94–97)	95 (91–96)	0.0005 *
**EtCO_2_ (mmHg)**	51 (44–59)	47 (42–53)	53 (44–61)	0.0090 *

* *p* < 0.05. C: Compliance, DP: Driving pressure, EtCO_2_: End-tidal carbon dioxide, FiO_2_: Fraction of inspired oxygen, I/E: Inspiratory-to-expiratory ratio, MP_dyn_: Dynamic mechanical power, MP_tot_: Total mechanical power, MVe: Minute ventilation, P/F: PaO_2_/FiO_2_ ratio, PaO_2_: Arterial oxygen pressure, P_mean_: Mean airway pressure, P_peak_: Peak airway pressure, P_plat_: Plateau pressure, PEEP: Positive end-expiratory pressure, RR: Respiratory rate, SpO_2_: Peripheral oxygen saturation, TV: Tidal volume, TV/PBW: Tidal volume normalized to predicted body weight, WOBv: Work of breathing.

**Table 3:** Analysis of laboratory parameters.

	All Patients (n: 310)	Survival (n: 76)	Non-Survival (n: 234)	*p*-Value
**WBC (×10^3^/µL)**	14.4 (9.9–20.2)	12.1 (9.3–14.7)	15.9 (11–21.4)	0.0002 *
**CK (U/L)**	178 (94–460)	299 (127–621)	157 (93–443)	0.0200 *
**proBNP (pg/mL)**	2370 (686–9189)	1750 (316–4750)	2463 (739–9620)	0.0521
**HCT (%)**	34 (30–39)	35(31–39)	34 (29–38)	0.1388
**Lym (×10^3^/µL)**	0.8 (0.5–1.2)	0.9 (0.6–1.2)	0.8 (0.50–1.2)	0.0820
**%Lym**	6.1 (4.0–8.7)	8.5 (5.9–12)	5.9 (3.7–8.2)	0.0001 *
**Neu (×10^3^/µL)**	12.7 (8.7–17.6)	10.2 (7.5–12.4)	13.9 (9.4–19.4)	0.0001 *
**%Neu**	88 (85–92)	86.2 (80.1–89.2)	89 (86–93)	0.0001 *
**Hgb (g/dL)**	10.8 (9.5–12.4)	11.2 (10.1–12.3)	10.5 (9.3–12.5)	0.0780
**Plt (×10^3^/µL)**	237 (159–306)	249 (186–322)	233 (151–306)	0.0770
**CRP (mg/L)**	129 (78–207)	121 (79–212)	130 (78–202)	0.4605
**Procalcitonin (ng/mL)**	1.6 (0.4–6.0)	0.8 (0.2–4.0)	1.7 (0.52–6.3)	0.0061 *
**LDH (U/L)**	540 (411–739)	481 (411–600)	585 (412–790)	0.0093
**D-dimer (µg/mL)**	2.4 (1.0–5.3)	2.8 (0.8–6.1)	2.4 (1.1–5.2)	0.4711
**Ferritin (ng/mL)**	822 (344–1843)	705 (328–1350)	837 (357–2108)	0.1898
**INR**	1.2 (1.1–1.4)	1.2 (1.1–1.3)	1.3 (1.1–1.4)	0.0109 *
**Creatinine (mg/dL)**	1.1 (0.6–2.0)	0.7 (0.5–1.0)	1.4 (0.7–2.2)	0.0001 *
**Fibrinogen (mg/dL)**	482 (359–655)	554 (348–700)	472 (360–634)	0.1591
**Albumin (g/L)**	27 (25–30)	28 (26–31)	27 (25–30)	0.0303 *
**Na (mmol/L)**	139 (134–142)	139 (136–143)	138 (134–142)	0.1020
**K (mmol/L)**	4.3 (3.7–4.8)	4.3 (3.9–4.6)	4.2 (3.9–4.9)	0.2176
**Cl (mmol/L)**	101 (97–105)	102 (99–104)	100 (97–105)	0.1599

* *p* < 0.05. CK: Creatine kinase, Cl: Chloride, CRP: C-reactive protein, D-dimer: D-dimer, Ferritin: Ferritin, Fibrinogen: Fibrinogen, HCT: Hematocrit, Hgb: Hemoglobin, INR: International normalized ratio, K: Potassium, LDH: Lactate dehydrogenase, Lym: Lymphocyte count, Na: Sodium, Neu: Neutrophil count, Plt: Platelet count, proBNP: Pro-brain natriuretic peptide, Procalcitonin: Procalcitonin, %Lym: Lymphocyte percentage, %Neu: Neutrophil percentage, WBC: White blood cell count.

## Data Availability

The data that support the findings of this study are available from the corresponding author upon reasonable request.

## Supplementary Materials

The following supporting information can be downloaded at: https://www.mdpi.com/article/10.3390/medicina61122086/s1, Table S1: Comparison of inflammatory biomarkers between patients below and above the driving pressure threshold (15.72 cmH_2_O); Table S2: The parameters examined in relation to the length of stay in the ICU.
