# Supplementary material for: Is Mechanical Power the Trojan Horse? Emphasizing the Role of Driving Pressure
Source: Medicina (Kaunas). 2025 Nov 22;61(12):2086. doi: 10.3390/medicina61122086 (PMC12734470; doi:10.3390/medicina61122086)
Supplement: Supplementary file 1 [file medicina-61-02086-s001.zip › medicina-3921356-supplementary.pdf]

Supplementary Digital Material

Inflammatory biomarkers were compared between patients above and below the driving pressure threshold. Among these markers, WBC and Lym% differed significantly between the groups. Patients with DP ≥ 15.72 cmH_2O had higher WBC values compared with those below the threshold (median [IQR]: 15.6 [11.8–21.3] vs. 13.0 [9.3–18.7], $p = 0.049$), along with a significantly lower Lym% (5.3% [3.6–8.4] vs. 7.4% [5.1–9.3], $p = 0.0069$). No significant differences were observed in CRP, Lym, procalcitonin, or ferritin levels between the groups (all $p > 0.05$) (**Table S1**).

Table S1. Comparison of inflammatory biomarkers between patients below and above the driving pressure threshold (15.72 cmH_2O)

	**DP < 15.72**	**DP ≥15.72**	***p***
**CRP (mg/L)**	121 (78-191)	137 (79-209)	0.64
**WBC (×10³/µL)**	13 (9.3-18.7)	15.6 (11.8-21.3)	**0.049**
**Lym (×10³/µL)**	0.88 (0.51-1.34)	0.82 (0.54-1.25)	0.52
**Lym %**	7.4 (5.1-9.3)	5.3 (3.6-8.4)	**0.0069**
**Prokalsitonin (ng/mL)**	1.08 (0.34-4.35)	1.58 (0.49-5.74)	0.362
**Ferritin (ng/mL)**	760 (354-1898)	845 (344-2046)	0.8

Several parameters demonstrated significant associations with ICU discharge timing. Higher driving pressure (HR: 1.85, $p = 0.0001$), elevated dynamic mechanical power (HR: 1.397, $p = 0.019$), increased plateau pressure (HR: 1.393, $p = 0.013$), and higher APACHE II (HR: 2.111, $p = 0.0001$) and SOFA scores (HR: 1.761, $p = 0.0001$) were associated with a greater hazard of ICU discharge, reflecting early mortality rather than faster clinical recovery. In contrast, lower BMI (HR: 0.595, $p = 0.0001$) and reduced PEEP (HR: 0.588, $p = 0.0001$) were associated with prolonged ICU stay. These findings indicate that ICU length of stay should be interpreted within the context of survival status, as shorter stays in

high-risk groups primarily reflect early death rather than improved outcomes (**Table S2**).

Table S2: The parameters examined in relation to the length of stay in the ICU

Covariate	Cut- off	HR	95 % CI	p-value
**P/F**	120.5	0.845	0.626-1.139	0.269
**Age (year)**	58	1.55	1.188-2.021	0.001
**Gender**		1.034	0.795-1.344	0.806
**BMI (kg/m²)**	25.1	0.595	0.452-0.784	0.0001
**$EtCO_2$ (mmHg)**	53	1.365	0.937-1.987	0.105
**RR (/min)**	14	1.373	0.991-1.903	0.057
**PEEP (cmH_2O)**	9	0.588	0.451-0.766	0.0001
**TV/PBW (mL/kg)**	8	0.768	0.570-1.035	0.083
**TV (mL)**	471	0.92	0.707-1.197	0.535
**MPtot (cmH_2O)**	18.6	1.249	0.956-1.631	0.103
**MPdyn (cmH_2O)**	10.08	1.397	1.057-1.847	0.019
**DP (cmH_2O)**	15.72	1.85	1.381-2.478	0.0001
**Ppeak (cmH_2O)**	25.4	1.111	0.839-1.470	0.464
**Pmean (cmH_2O)**	16.3	0.82	0.630-1.066	0.138
**Pplateau (cmH_2O)**	25.7	1.393	1.073-1.808	0.013
**SpO_2 (%)**	94	0.953	0.735-1.237	0.72
**PaO_2 (mmHg)**	85	0.968	0.736-1.273	0.815
**APACHE II**	23	2.111	1.607-2.774	0.0001
**Sofa**	10	1.761	1.318-2.353	0.0001

* $p < 0.05$.

APACHE II: Acute Physiology and Chronic Health Evaluation II, BMI: Body mass index, CI: Confidence interval, DP: Driving pressure, $EtCO_2$: End-tidal carbon dioxide, HR: Hazard ratio, MPdyn: Dynamic mechanical power, MPtot: Total mechanical power, PaO_2: Arterial oxygen pressure, PBW: Predicted body weight, PEEP: Positive end-expiratory pressure, P/F: PaO_2/FiO_2 ratio, Pmean: Mean airway pressure, Ppeak: Peak airway pressure, Pplat: Plateau pressure, RR: Respiratory rate, SOFA: Sequential Organ Failure Assessment, SpO_2: Peripheral oxygen saturation, TV: Tidal volume, TV/PBW: Tidal volume normalized to predicted body weight.
